# Identification of critical paralog groups with indispensable roles in the regulation of signaling flow

**DOI:** 10.1038/srep38588

**Published:** 2016-12-06

**Authors:** Dezso Modos, Johanne Brooks, David Fazekas, Eszter Ari, Tibor Vellai, Peter Csermely, Tamas Korcsmaros, Katalin Lenti

**Affiliations:** 1 Department of Morphology and Physiology, Faculty of Health Sciences, Semmelweis University, Budapest, Hungary; 2 Department of Genetics, Eotvos Lorand University, Budapest, Hungary; 3 Earlham Institute, Norwich Research Park, Norwich, UK; 4 Gut Health and Food Safety Programme, Institute of Food Research, Norwich Research Park, Norwich, UK; 5 Faculty of Medicine and Health, University of East Anglia, Norwich, UK; 6 Department of Gastroenterology, Norfolk and Norwich University Hospitals, Norwich, UK; 7 Synthetic and Systems Biology Unit, Institute of Biochemistry, Biological Research Centre of the Hungarian Academy of Sciences, Szeged, Hungary; 8 Department of Medical Chemistry, Semmelweis University, Budapest, Hungary

## Abstract

Extensive cross-talk between signaling pathways is required to integrate the myriad of extracellular signal combinations at the cellular level. Gene duplication events may lead to the emergence of novel functions, leaving groups of similar genes - termed paralogs - in the genome. To distinguish critical paralog groups (CPGs) from other paralogs in human signaling networks, we developed a signaling network-based method using cross-talk annotation and tissue-specific signaling flow analysis. 75 CPGs were found with higher degree, betweenness centrality, closeness, and ‘bowtieness’ when compared to other paralogs or other proteins in the signaling network. CPGs had higher diversity in all these measures, with more varied biological functions and more specific post-transcriptional regulation than non-critical paralog groups (non-CPG). Using TGF-beta, Notch and MAPK pathways as examples, SMAD2/3, NOTCH1/2/3 and MEK3/6-p38 CPGs were found to regulate the signaling flow of their respective pathways. Additionally, CPGs showed a higher mutation rate in both inherited diseases and cancer, and were enriched in drug targets. In conclusion, the results revealed two distinct types of paralog groups in the signaling network: CPGs and non-CPGs. Thus highlighting the importance of CPGs as compared to non-CPGs in drug discovery and disease pathogenesis.

The cellular signaling system relays information between the external and internal milieus of the cell and helps to adapt to the varying microenvironment. Based on incoming signals, cells make decisions such as whether to proliferate, change metabolism, secrete various proteins or molecules, differentiate, or die^1^. Incoming signals are channeled by a few signaling pathways, which are both evolutionarily conserved and biochemically different^2^. To ensure an appropriate response, the signaling system maintains the output specificity of the pathways (inputs preferentially activate their own output) and input fidelity (outputs preferentially respond to their own input)^3^. Malfunctions in signal transduction can cause major system-level diseases such as cancer, diabetes, or neurodegenerative disorders^4^.

However, a limited number of pathways alone cannot adequately respond to the myriad of different combinations of incoming signals. Thus, inter-pathway connections are required for the cells, which are called *cross-talks*. During evolution, cross-talks have been formed and changed more frequently than signaling pathways themselves^5^,^6^. Novel cross-talks could also emerge as a result of evolutionary gene duplications of signaling pathway members^7^. These duplication events may allow one of the duplicates to develop partially different functions like cross-talk with other pathways while the other duplicate maintains the original function and original flow of information^7^,^8^,^9^. Related genes, which have emerged from a gene duplication event within a single genome are termed *paralogs*. Since their duplication event paralogous genes diverge from each other by sequence alterations, which serve as an important mechanism in the emergence of their differing functions. Thus, paralogs are likely but not necessarily diverged in their function. Importantly, paralogs participating in signaling mechanisms could form novel cross-talks between signaling pathways.

In this work, we aimed to find paralog groups in the human cellular signaling pathways that participate in cross-talks and have an important role in the signaling flow. To define what is an important element in the signaling flow, we used the graph theory based term ‘criticality’^10^,^11^. In graph theory a critical node is known as a vertex, whose removal results in the dissociation of the original graph to multiple subgraphs or the disconnection between source vertices (i.e. those vertices, which have outgoing edges only) and sink vertices (i.e. vertices having incoming edges only)^10^,^11^,^12^. We built our analysis on the latter definition and adapted it for signaling pathways, thereby identifying source nodes as ligands, and sink nodes as transcription factors (TFs), and connections between them as directed tissue-specific paths from ligands to TFs.

We defined *critical paralog groups* (CPGs) using three criteria: (1) CPGs are a group of proteins which were formed from paralogous genes (*evolutionary criterion*), (2) at least one member of the group forms a cross-talk between at least two signaling pathways (*biological criterion*), (3) and at least one member of the group connects a ligand to a transcription factor in a signaling path in a given tissue (*tissue-specific signaling criterion*) (Fig. 1a). With these criteria our method is capable of distinguishing critical paralog groups from the non-critical paralog groups in signaling networks whereas non-critical paralog groups are deficient in at least one of the above three criteria. The method was tested using the following hypothesis: CPGs may have a more important role in the development of cancer and other systems diseases than non-critical paralog groups.

We note that the definition of CPGs is a modification of Kahn and colleagues’ definition of critical nodes that they applied in the insulin signaling pathway^8^, where the members are (1) essential in the signal transduction of a given pathway, (2) related to each other (paralogs), (3) regulated and functioning in a partially different way and (4) at least one of the members participate in cross-talk with another pathway. Our critical paralog group approach extends Kahn and colleagues’ definition to multiple signaling pathways and is more universally defined as a tissue-specific graph based criterion. With the described biological-, evolutionary- and graph-based criteria, we identified critical paralog groups that are important in signaling networks and distinguished them from duplicated genes, which have less significance in signaling processes.

## Materials and Methods

### Source of signaling pathway data

The human signaling pathway data, including the lists of proteins from seven distinct signaling pathways and together with their interactions, were obtained from the SignaLink 2 database^6^,^13^. All seven pathways – the RTK/MAPK (receptor tyrosine kinase/mitogen-activated protein kinase), TGF-β (transforming growth factor-beta), Notch, WNT/Wingless, Hedgehog, JAK/STAT (Janus activating kinase/signal transducer and activator of transcription), and NHR (nuclear hormone receptor) – available in SignaLink 2 were examined in this study. SignaLink 2 database^13^ was chosen because it sorts human signaling pathways based on their different evolutionary origin, contains directed and traceable interactions between proteins and – compared to other resources – it incorporates a high amount of cross-talk between pathways (Turei *et al*. personal communication^14^). It also contains the functional role of proteins in signaling pathways, such as ligands, or transcription factors. All features above were essential to perform the identification of CPGs. To the best of our knowledge, other pathway resources do not collect all of these characteristics. A protein was labeled as being involved in cross-talk if it was connected to a protein participating in a different signaling pathway.

### Data sources for the identification of paralog groups

Our aim was to form paralog groups containing the highest amount of proteins listed in SignaLink 2. We defined paralog groups as homologous human proteins based on the clustering of OrthoDB and InParanoid resources^15^,^16^. OrthoDB uses best reciprocal hit between two genomes, thus it finds orthologs (similar genes *between* species). Then OrthoDB searches for paralogs (similar genes *within* a genome) in the query genomes separately that are more similar than the found orthologs^15^. We created paralog groups based on mammalian genes similar to the human genes. The mammalian ortholog file, which contains the above described similarities (“ODB8_EukOGs_genes_Mammalia-40674.txt”), was downloaded on 2^nd^ November 2015 from OrthoDB.

InParanoid makes pair wise BLAST searches between two genomes and forms connections between genes^16^. Thus, using InParanoid, we defined paralog groups by pair wise searches of paralogs and orthologs in all mammalian species that are connected with each other. We downloaded the results of pair wise searches of all mammalian species from InParanoid database on 10^th^ October 2015. We then constructed a graph where the nodes were the orthologues in different species and the edges were the pair wise similarities between them. Human proteins connected by a path of pair wise similarities were considered to be part of the same paralog group. Thus, we extracted paralog groups from OrthoDB, and constructed graph based paralog groups from InParanoid, where the edges were pair wise similarities.

Next, the two complementary sources were merged to maximize the coverage with SignaLink 2. We aimed to construct exclusive paralog groups with fewer members that reflect more specific similarities within each group and more differences between the groups. To do this we constructed distance metrics, which measure the amount of SignaLink 2 proteins and the ratio of SignaLink 2 proteins in the paralog groups. See Equation 1, where *D* is the distance measure, *n* is the number of proteins in the paralog group and *m* is the number of proteins of paralog groups.


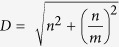


We used the highest *D* metric to address a protein to particular group. If a protein appeared in multiple similar *D* scored paralog groups, the protein was annotated to the paralog group that contained the highest amount of SignaLink 2 protein (*n*).

### Further resources

Tissue expression data were obtained from the ‘egenetics’ anatomical location expression source of the Ensembl v74 resource^17^. As we found the grouping of tissues too detailed (listing 135 tissues separately), we combined similar tissues to 18 main organ systems (Suppl. Table 1).

To measure the potential pathogenic effects of malfunctioning proteins in different groups, the number of corresponding gene alleles causing inherited diseases or functioning as driver gene mutations in cancer were counted. We also enumerated the number of diseases belonging to each protein’s gene. Cancer-causing driver mutation data were collected from Cancer Gene Census (downloaded on 11^th^ November 2015), which is a part of the COSMIC database^18^. We used the OMIM database^19^ to ascertain whether a mutation of a protein coding gene is contributing in inherited diseases or not (data were downloaded on 14^th^ December 2015). To analyze the drug discovery relevance of examined proteins we downloaded the currently used drug targets from ChEMBL (version 20)^20^.

To obtain transcriptional regulatory information for each protein, we used experimental data from Oreganno^21^ (accessed through the PAZAR^22^ resource on 2^nd^ November 2015) and HTRIdb resources^23^ (downloaded on 9^th^ October 2015). For miRNA target identification, we used the experimentally verified data from the miRTarBase database^24^ (downloaded on 9^th^ October 2015). For functional annotations, the Biological Process and Molecular Functions domains of Gene Ontology (GO)^25^ database were used (downloaded from UniProt database^26^ on 9^th^ October 2015).

### Methods and network parameters for the analysis of critical paralog groups

To analyze the importance of each protein in signaling networks we measured their *node degree* (number of neighbors), *betweenness centrality* (number of shortest paths going through a certain node), *bowtieness* (percentage of shortest paths from a ligand to a transcription factor going through a given protein^27^), and *closeness* (reciprocal mean distance of a given node from all other nodes^28^). Node degree measures the local importance of a node while, betweenness reflects its global importance. Bowtieness is a similar measurement to betweenness but more specific to signaling networks^27^. Closeness measures whether the node is in the core of the network (high closeness) or at the periphery, far from other nodes (low closeness). Different network parameters were measured by Igraph in Python environment^29^. The network analysis figures were made with “vioplot” R package^30^.

### Specificity analysis

To compare the similarity within paralog groups, a simple regulatory, functional and disease similarity metric were constructed to evaluate whether a critical paralog group (CPG) has more or less similar features than a non-critical paralog group (non-CPG) within a paralog group. To do this, we counted the ‘specific features’ of each protein within a group. We termed a feature *specific* if not all of the members of the group had that particular feature. Then we divided the number of specific features within the group by the number of proteins of the group to normalize it to the size of the group. Throughout the manuscript we are using the term ‘specific’ in the context defined here.

### Constructing tissue-specific networks

Tissue classification (described previously, Suppl. Table 1) was applied to assign each SignaLink 2 protein to one or multiple tissues. For each tissue, we constructed a tissue-specific network if more than half of all SignaLink 2 proteins were assigned to that tissue. If a protein was not assigned to a particular tissue it was ruled out from a tissue-specific graph (165 out of the 733 SignaLink 2 proteins). To control this effect, every tissue-specific graph was created with and without the unannotated proteins. For both kinds of tissue-specific networks, all possible paths between ligands and transcription factors were measured. If we lost a path by removing a given protein in any tissue, then we declared the given protein as essential to that path. We conducted the analysis separately with the unannotated proteins both present and absent.

### Statistical evaluation

To test the differences between categorical variables, like drug relatedness or mutations in cancer, chi-square tests were applied. In all evaluations, we compared the examined parameters to the whole database, which contained all proteins from SignaLink 2 and their paralogs. For statistical tests of nominal variables, which did not have a normal distribution, like network centralities, we used both the two sample Kolmogorov-Smirnov test and Wilcoxon rank sum test. As all significance values were the same for statistical tests of nominal variables, we list only the *p*-values of the Wilcoxon rank sum test in the text since it is more rigorous. Kolmogorov Smirnov tests gave the same results. For gene ontology enrichment analysis *p*-values were calculated using the hypergeometric tests and corrected for multiple testing by the Benjamini-Hochberg algorithm^31^. We used all SignaLink 2 proteins and their orthologues in the analysis as background. The statistical evaluations were computed in R enviroment^32^.

## Results

### Identification of critical paralog groups (CPGs)

**Figure 1:**
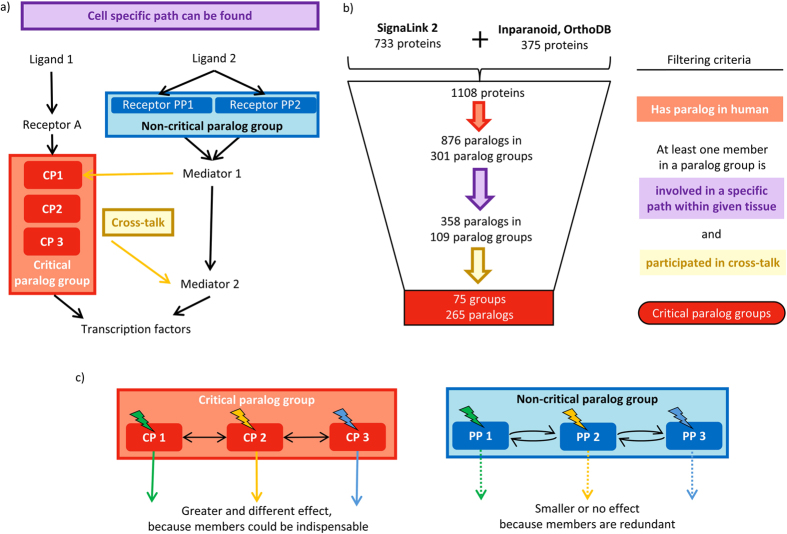
Definition and identification of critical paralog groups. (**a**) Definition of critical paralog groups. A critical paralog group contains critical paralogs (CP) that are paralogs of each other have at least one unique path through them in a specific tissue and have at least one cross-talking member. (**b**) The workflow of identifying critical paralog groups. (**c**) Effect of diseases such as cancer and drugs in CPGs and in non-CPGs. Targeting a paralog protein will have no effect, because another protein from the same group could maintain its function, but targeting a CP will cause an effect, because CPs within a CPG are indispensable. See details in the main text.

We constructed a workflow to identify critical paralog groups and critical proteins as follows (Fig. 1b):

1. **Construction of paralog groups:** First we searched for the paralogs of the 733 human signaling proteins of SignaLink 2 in InParanoid and OrthoDB resources, and identified paralog resources as described in the Materials and Methods. This resulted in 301 paralog groups containing 876 proteins.
2. **Criticality in the signaling flow:** Using tissue-specific signaling networks we selected 109 paralog groups (from the original 301 paralog groups) that contained at least one protein per group that connects a ligand to a transcription factor in a given tissue and without which the signal cannot reach the transcription factor in a given tissue. Altogether the 109 paralog groups contained 358 signaling proteins.
3. **Cross-talking groups:** Combining the cross-talk information (interaction between two proteins from different pathways) of SignaLink 2 database with the 109 paralog groups, we selected 75 groups that had at least one cross-talking member.

Based on these three filtering steps 75 critical paralog groups (CPGs) were determined in the human signaling network that contained altogether 265 critical paralogs (CPs). Among these 265 CPs 168 proteins were found directly in SignaLink 2 and the additional 97 proteins were paralogs of the SignaLink 2 proteins. For the full list of CPs, see Suppl. Table 2. For further analyses we divided the other, non-critical proteins into two groups: (1) Proteins that had paralogs but their group members were not critical or did not form cross-talks. The groups themselves were termed as ‘non-critical paralog groups’ (non-CPGs) and the proteins in non-critical paralog groups were termed as ‘paralog proteins’, PPs (Fig. 1c). There were altogether 226 non-CPGs containing 611 PPs. (2) SignaLink 2 proteins without any paralogs (232 cases) were simply termed as ‘other proteins’.

### Critical paralogs in the human signaling network

**Figure 2:**
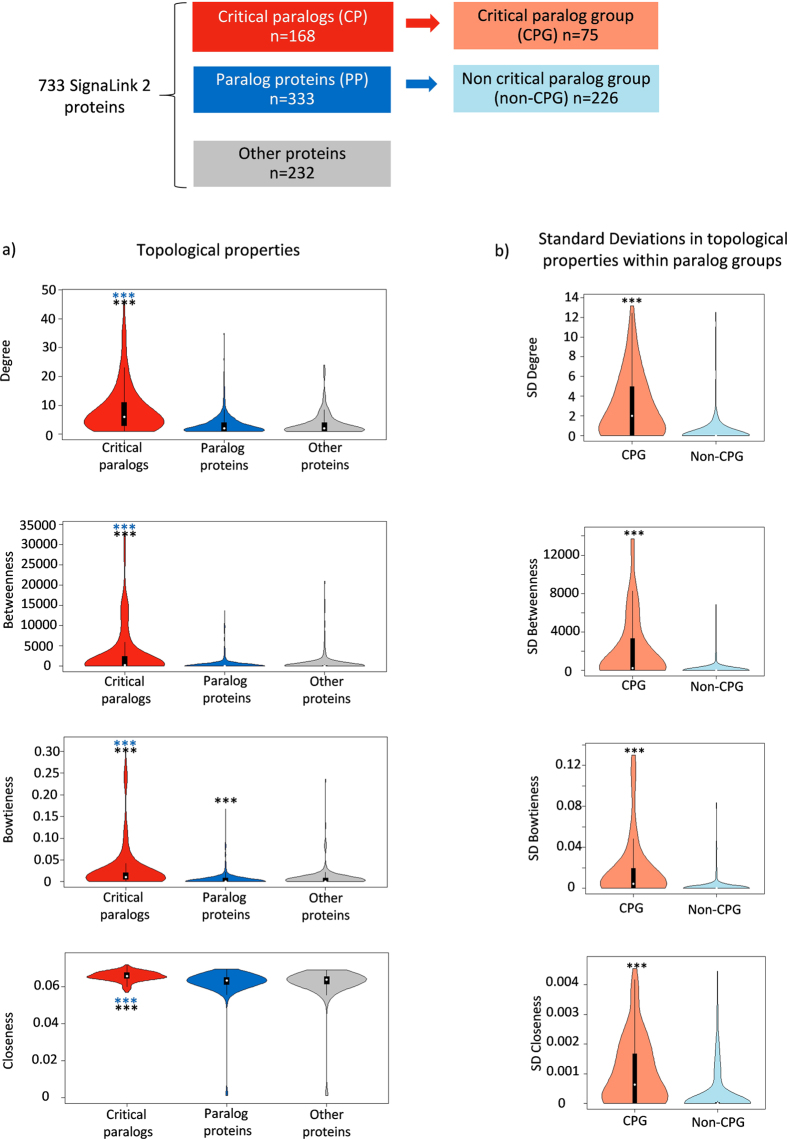
Network parameters of critical paralog group proteins. (**a**) Values of network parameters in different groups of proteins. (**b**) Standard deviations of network parameters within each group. ***shows where *p* < 0.001 in the Wilcoxon rank sum tests, blue stars stand for significant difference between critical proteins and paralog proteins (PPs), black stars stand for significant difference between critical proteins or PPs and other proteins.

We examined four network parameters, degree, closeness, betweenness centrality and bowtieness to demonstrate the important topological role of CPs in the signaling networks (see Materials and Methods for details). We found that CPs have significantly higher values of degree, closeness, betweenness centrality and bowtieness compared to PPs or other proteins. (Wilcoxon rank sum test, *p *< 0.001, Fig. 2a). A higher degree (more connections) represents local importance and potential involvement in more biological functions^33^. Higher betweenness centrality means CPs are more important for the global communication of the network since many of the shortest signaling paths are going through them. Higher bowtieness represents higher importance in signaling function. Higher closeness shows that the CPs form the central part of the network. Thus, all examined centrality measurements showed a consistent higher importance of CPs compared to PPs and other proteins. Higher centrality in protein interactions or signaling networks could implicate involvement in additional biological functions so loss of important proteins could be lethal or could lead to developmental arrest: they are essential in genetic terms^28^.

We measured not only the values of centrality network parameters but also the parameters’ standard deviation within a paralog group. We found that the CPGs have higher standard deviations within their groups compared to non-CPGs (Fig. 2b). The higher standard deviation within a CPG indicates the diverse role of CPs within even one CPG to connect different paths in the signaling network. Higher standard deviation suggests that CPs are indispensable within a CPG; CPs have different network parameters compared to each other within the same CPG. The higher centrality measurements of CPs and the higher diversity of the CPs in a CPG showed indispensable network function of critical proteins and their groups in the signaling network.

### Specific regulation and more diverse function in critical paralog groups

**Figure 3:**
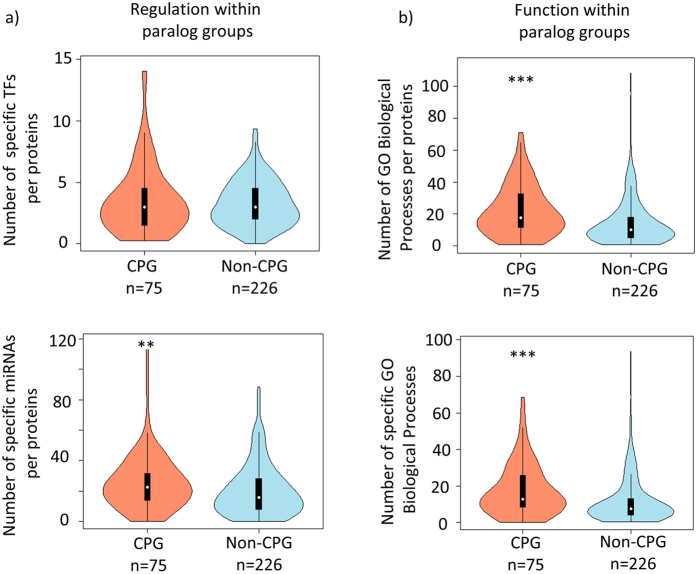
Regulation and function of critical paralogs. (**a**) Number of transcription factors and miRNAs that are specific to a member protein in the critical paralog groups (CPG) and in non-critical paralog groups (non-CPG). (See Materials and Methods for the definition of the term “specific”.) (**b**) Number of all and specific Gene Ontology Biological Processes terms per protein in CPG and non-CPG. **shows where *p* < 0.01, and ***shows where *p* < 0.001 in the Wilcoxon rank sum test.

According to our data, almost all paralog groups (294 paralog groups out of 301) had partly different regulation by transcription factors or miRNAs – i.e. at least one transcription factor or miRNA does not regulate all members of a paralog group. Thus, we analyzed the specificity and differences of the regulation between CPGs and non-CPGs. We found that the transcriptional regulation was not different between CPGs and non-CPGs, i.e. the number of specific transcription factors per protein was statistically similar (see Materials and Methods for details). Thus, on one hand, transcriptionally CPGs were not regulated more diversely than non-CPG-s. On the other hand, the post-transcriptional regulation of CPGs was more specific than that of non-CPGs. There were more paralog-specific miRNAs to down-regulate a particular member of a CPG’s expression than paralog-specific miRNAs to downregulate the expression of a particular member of non-CPG (Fig. 3a).

Not all the paralog proteins from a paralog group have been annotated to a pathway. 97 critical paralogs in critical paralog groups and 278 paralog proteins in non-critical paralog groups were not present in the SignaLink 2 database. Other, annotated members of the paralog group could help the annotation of these proteins in signaling pathways. Some of these proteins could not even be annotated to any Gene Ontology biological process. 9 critical proteins and 30 paralog proteins were such unannotated proteins. We believe these un-annotatable proteins do not introduce bias to our analysis, since they possess the same abundance in critical paralogs and paralog proteins showing no significant difference (Chi square test, *p *= 0.309).

CPGs were connected to more Gene Ontology Biological Processes terms per proteins than non-CPGs. (Fig. 3b). The functions of CPs are more specific for each CP in each CPG than PPs within a paralog group (Fig. 3b, see Materials and Methods for details). Thus, critical proteins within a CPG do not just have a larger spectrum of topological importance (Fig 2b), they are also extensively involved in different Biological Processes. The diverse and paralog-specific functions within a CPG require specific regulation. This role may be fulfilled by post-transcriptional regulation through miRNAs, the fine tuners of gene expression.

We also investigated the Gene Ontology Molecular Functions of critical proteins. We found CPs are more often transcription factors (*p < 0.001*), and also bind more often to type I interferon receptors (*p < 0.001*) and I-SMADs (*p = 0.0453*) – mediators of JAK-STAT and TGF-beta pathways respectively – when compared to all proteins in the analysis (as a background). For the full list of enriched Molecular Functions of CPs see Suppl. Table 3. All signaling information culminates in the activation of transcriptional factors. Importantly, CPs are enriched at the downstream part of signaling, among transcription factors which provides the last step before gene expression changes occur. (Suppl. Table 3).

### Medical and pharmacological relevance of the critical paralog groups

**Figure 4:**
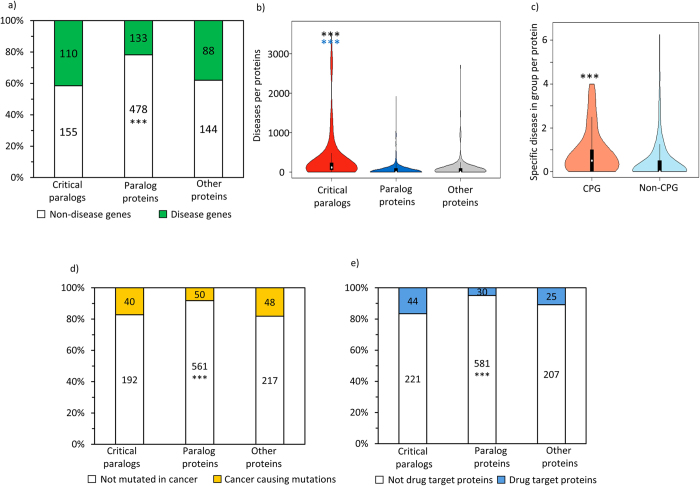
Critical paralog groups in disease and drugs. (**a**) Percentage of diseases in critical paralogs, paralog proteins, and in other proteins. (**b**) Disease per protein in critical paralogs, paralog proteins and other proteins, (**c**) Specific diseases per protein in CPGs and in non-CPGs. (See Materials and Methods for the definition of the term “specific”.) (**d**) Occurrence of cancer-causing genes in critical paralogs, paralog proteins and other proteins, (**e**) Occurrence of drug targets in critical paralogs, paralog proteins and in other proteins. To calculate differences between categorical variables we used Chi-square tests (**a,d,e**). For continuous variables (**b,c**) we used the Wilcoxon rank sum test. (***shows where *p* < 0.001) Blue stars stand for significant difference between CPs and paralog proteins, black stars stand for significant difference between CPs or paralog proteins and other proteins.

Finally, we analyzed the disease relevance of critical paralog groups. We investigated whether mutation of CPs contributed more often to inherited diseases. We found that CPs had a similar abundance of genes involved in heritable diseases as other proteins (41% vs. 37%, Chi square, test *p *> 0.05). On the contrary, PPs had a significantly smaller abundance of genes involved in heritable diseases than other proteins (21% vs. 41% or 37%, Chi square test, *p *< 0.001; Fig. 4a). There were more inherited diseases per one single protein among CPs than PPs or other proteins (Wilcoxon rank sum test, *p *< 0.001) demonstrating that CPs are involved in multiple pathological conditions (Fig. 4b). We also examined whether critical proteins from the same CPG cause the same disease or contribute to different disorders. To do that we performed the same specificity analysis of diseases in a paralog group what we had made with GO functions and regulations (see Materials and Methods for details). We found that critical proteins from the same CPG do not cause the same diseases. However, PPs from the same non-CGP were involved in the same diseases (Wilcoxon rank sum test, *p *< 0.001; Fig. 4c). The observed similar ratio of disease causing genes between CPs and other proteins means the function of CPs and other proteins were equally non-redundant in signaling. PPs had redundant functions (Fig. 3a), so a potential disease causing effect of a mutation could be at least partly rescued by another paralog protein from the same non-CPG (Fig. 1c).

We also measured the occurrence of cancer-causing (driver) mutations. According to literature, cancer targets are topologically the most central proteins in protein-protein interaction networks^34^. Similarly, CPs are central proteins – as we have shown earlier in this study (Fig. 2a). In accordance with this result, we found more CPs involved in cancer development than PPs (17.2% vs. 8,2%, Chi square test, *p *< 0.001). Similar to the previous analysis, we found that mutations in PPs were less often drivers than in either CPs or in other proteins (Fig. 4d). CPs and other proteins in signaling could be indispensable in signaling; meanwhile PPs from the same non-CPG could substitute each other’s function (Fig. 1c).

The high importance of critical proteins in signaling taken with their wide range of biological functions and high occurrence in cancer and other diseases makes them potential drug targets as they can have a broad effect on cells^35^. Therefore, we measured how often CPs present as drug targets in the ChEMBL database. We found that CPs are more enriched in drug targets than PPs (16% vs. 4%, Chi square test, *p *< 0.005; Fig. 4e). Critical proteins are indispensable but PPs are similar to each other (Fig. 1c). Therefore, if a PP is targeted, more than one drug target might be necessary to achieve a significant pharmacological effect (Fig. 1c). On the contrary, targeting CPs is more effective, since they are indispensable but require paralog-specific drugs and careful selection. CPs hold a central position within the network (Fig. 2a) and have diverse functions (Fig. 3b) so targeting such proteins could lead unpredictable side effects^35^,^36^.

### Examples of critical paralog groups

**Figure 5:**
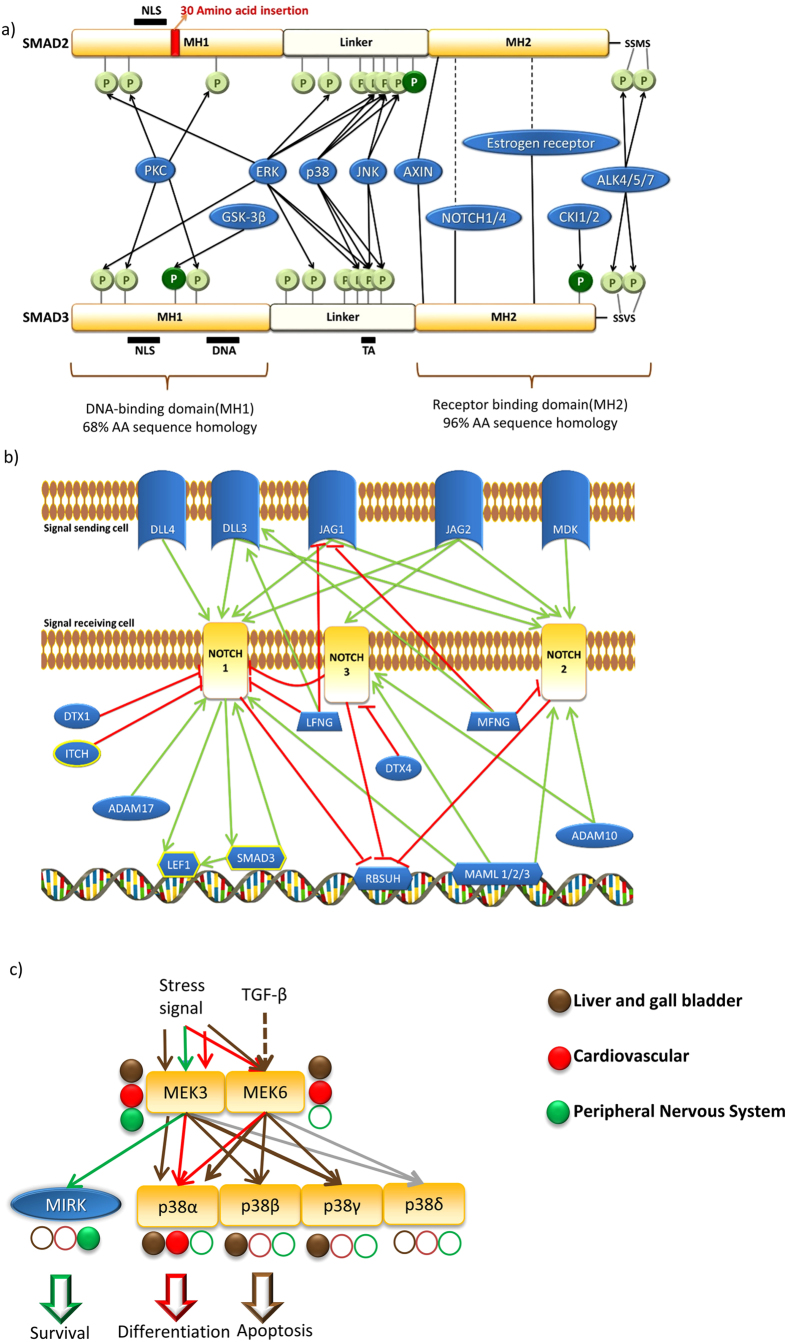
Three examples of critical paralog groups. Each example shows a major mechanism that makes them a source of signaling diversification: paralog-specific interaction profiles, expression patterns, and protein regulators. (**a**) SMAD2 and SMAD3 critical paralog proteins are represented with their domain structures and major phosphorylation motifs. Note the 30 amino acid insert in SMAD2 (red), the different phosphorylation sites (dark green) and the different binding partners of SMAD2 and 3, including the absence of the DNA-binding site in SMAD2. Only multi-pathway protein interactors are presented (except ALK4, ALK5 and ALK7, which are receptors of the TGF-β pathways). Phosphorylations are represented by directed edges from a kinase, and protein interactions by edges without arrows. Relative affinity of an interaction is shown with normal and dashed lines. NLS: Nuclear Localization Signal, TA: Transcription Activator. Structure and binding information are based on the work of Wrighton *et al*.^41^. (**b**) The role of paralog-specific ligands and co-factors is demonstrated with the NOTCH critical paralog group. Activating interactions are shown with green arrows, inhibiting interactions with red blunted arrows. Dashed links are for indirect effects. NOTCH interactors are shown in blue, and those that function in other pathways are highlighted with a yellow border. Only NOTCH1 has connections to such proteins. MFNG and LFNG have opposing effects on NOTCH1 and NOTCH2 meanwhile, NOTCH3 can inhibit NOTCH1 interactions within the Notch pathway. (**c**) Two MAPK critical paralog groups. For clarity, only selected interactor proteins are shown, and we excluded phosphatases and scaffold proteins. We present the expression patterns of each protein in three selected tissue types where the expression difference illustrates its regulatory role in the regulation of signaling flow: liver and gall (brown), cardiovascular (red), peripheral nervous system (green). Full circles represent expressed proteins, while empty circles represent non-expressed ones. Edges are marked with the color of the tissue, where the given interaction may happen as both interactor proteins are present. Note, the different ways of signaling flow and output functions in the three tissues based on expression levels only.

The more diverse network centrality and biological function distributions of CPGs suggest that CPGs have evolved novel phenotypical traits and novel biological functions through novel signaling cascades. One such example is the SMAD2 and 3 pair, the two major mediators of TGF-β pathway. (Fig. 5a) They form a double heterodimer complex in response to TGF-β and transduce the signal to the nucleus^37^. 90% of the amino acid sequences of SMAD2 and SMAD3 are identical. The gene encoding SMAD3 was duplicated in the genome during evolution^38^. After this duplication event, the ancient form of the SMAD2 gene was probably freed from selection pressure and so accumulated a 30 amino acids insertion in its MH1 domain that caused a structural change^38^. Compared to SMAD3, SMAD2 is unable to bind DNA and it has partially different protein binding partners^39^,^40^ (Fig. 5a). Both proteins have cross-talks with the WNT and RTK/MAPK pathways, as well as paralog-specific cross-talks to the Notch and NHR pathways (Fig. 5a)^6^,^41^. They are important in embryonic differentiation and in regulation of the epithelial-mesenchymal transformation, from which malfunctions can lead to malignancy^19^. SMAD2 is responsible for the formation of the dorsoventral axis, while SMAD3 can negatively regulate the cell cycle and can induce apoptosis^26^. To induce TGF-β-specific gene expression changes in response to a TGF-β signal, both members of the SMAD2 and 3 pair have to be present^37^. If only one member of the pair is present (e.g., either SMAD2 or SMAD3) or their relative concentration differs significantly, then the TGF-β signal can influence other pathways through the cross-talk of the dominant paralog. For example, TGF-β can activate the WNT signaling through SMAD3 and SMAD3 can inhibit the expression of AXIN, a negative regulator of the WNT pathway^42^. This cross-talk is important in the maturation of chondrocytes^42^. So, the SMAD2 and SMAD3 pair is a prime example of how a structural difference (e.g., insertion of a short amino acid sequence affecting one paralog’s DNA binding capability) can lead to divergence and variations in biological functions and cross-talks.

CPGs representing novel evolutionary traits require novel and strict regulation. One possibility of such regulations is the different co-factors targeting different CPs in a CPG. A good example of this phenomenon can be found in the Notch pathway. Here, three paralogs of the denominator NOTCH protein form a CPG, which contains NOTCH1, NOTCH2 and NOTCH3. This pathway is distinct as the NOTCH proteins are integrated receptor, mediator, and transcription factor proteins. Upon activation, the NOTCH receptor is cleaved and enters the nucleus where it can alter gene expression^43^. As all three NOTCH paralogs are expressed ubiquitously in nearly all tissues, only the relative concentration of each paralog and the paralog-specific ligands (e.g., DLL3/DLL4) or co-factors (e.g., DTX1/DTX4, LFNG/MFNG) can determine the dominant NOTCH paralog within this CPG. Mutation in different NOTCH paralogs, just like in many other CPGs (Fig. 5c), causes different diseases demonstrating that the NOTCH paralogs have (partially) distinctive biological functions. Mutation of NOTCH1 for instance causes a bicuspid aortic valve^44^, mutation of NOTCH2 leads to the liver disease Alagile syndrome^45^, whereas mutation of NOTCH3 causes insufficient vascular development in the brain^46^. Interestingly, only the NOTCH1 paralog has been shown to cross-talk with other pathways; it can cross-talk via LEF1 with the WNT pathway and it shows bidirectional cross-talk with SMAD3 of the TGF-β pathway^42^,^47^ (Fig. 5b). In the NOTCH-related CPG, the three members can influence each other. For example, NOTCH3 can inhibit the Notch pathway-specific transcription activity of NOTCH1, while it does not affect the cross-talk activity of NOTCH1^48^. The three NOTCH proteins are a good example of a CPG, whose members have similar molecular functions, but different co-factors and cross-talk options can dramatically shift their functions.

The importance of expression-based regulation of signaling flow can be seen with a subset of the MAPK pathway (Fig 5c). It is well-known that the MAPK pathway is formed by several paralog groups^49^. The inherent combination possibilities of the MAPK pathway are regulated by paralog-specific phosphatases, scaffolds, feedback-loops and tissue-specific co-expressions^1^,^49^,^50^,^51^. Here we focus only on the importance of tissue-specific co-expression in two MAPK-related CPGs. This illustrates the effect of tissue-specific gene expression regulation in determining the signaling flow and biological output response (Fig. 5c). MEKs are kinases and members of the so-called mitogen activated kinase kinases (MAP2K) family. MEKs phosphorylate ERK type kinases, such as the p38 paralog group^52^. MEK3 and MEK6 paralogs receive stress related signals^53^, meanwhile MEK6 also accepts signals from the TGF-β pathway^54^. MEK3 and MEK6 have no known target specificity in p38 paralogs (p38α, p38β, p38γ, p38δ) without scaffolding. The final effect depends on the tissue-specific expression of p38 paralogs. If only p38α is expressed, then the MEK3/6-p38 path induces specific myogenic differentiation that occurs in cardiac myoblasts^55^. In the liver, activation of p38β through MEK6 is a known TGF-β induced apoptotic pathway^54^ and can also lead to hepatocellular fibrosis^56^. In the peripheral nervous system, no p38 CPG proteins are expressed so the incoming signals from MEK3 can only reach a kinase called MIRK. This activation is known as an important survival signal in the peripheral nervous system^57^. Consequently, the expression differences within a CPG can determine the functional outcome of a transduced signal: ‘life’, ‘differentiation’ or ‘death’. In the p38 CPG, specific expression of members can define the biological response to similar incoming signals in different tissues: survival in the peripheral nervous system, differentiation in the heart, and apoptosis in the liver.

In drug discovery members of the ‘EGFR’ CPG (EGFR1, HER2, ERBB3, ERBB4) are current examples in targeting critical paralogs. These are relevant in several cancer types and are targeted by specific antibodies in metastatic colon cancer^58^ and breast cancer^59^. Targeting all members of the CPG is also a clinically relevant strategy, as shown by the small molecule, afatinib, which also blocks ERBB4 as well as EGFR and HER2^60^. There are also CPGs that are not (yet) drug targets. This might be because these CPGs are ‘difficult’ targets given their high centrality in the network, which may cause a large amount of side effects^35^. One such example is the previously discussed SMAD2 and SMAD3 CPG, where SMAD3 has the highest degree from proteins in the SignaLink 2 network. Targeting the neighborhood of critical proteins may overcome the issues of specificity and side effects^61^. Meanwhile, other not-yet described drug target CPGs, like receptors of immune functions, may also be notable candidates for pharmaceutical research. For example, the ‘OSMR’ CPG (OSMR, LIFR, CRLF1) is a member of the JAK-STAT pathway. Members of the ‘OSMR-CPG’ transmit a cytokine response through GP130^62^,^63^ and via JAK1 they also show cross-talk with the MAPK pathway^64^. OSMR is already a drug target candidate against cervical squamous cell carcinoma^62^. Targeting one CP in a CPG can help to rewire a signaling path to cure malignant diseases, as we have seen above in case of the EGFR and OSMR CPGs. Also in such cases careful selection is required, because too central a target may have wide-spreading unpredictable effects in the cell similar to the target SMAD3. As an important addition, we note that most of these CP drug targets were found by screening assays and network centrality based identifications whereas in this study we provide a *systematic workflow* to identify these pharmacologically relevant proteins and their potential signal modulating functions.

## Discussion

Signaling networks specifically transduce a large variety of signals with a limited number of pathways. Regulation of signaling flow within and between pathways is a key process in developmental biology and biomedicine. Identifying critical parts of signaling is a key issue of network biology^28^. Previous studies used network based^65^,^66^,^67^ and biological measures alone^8^ or combined^68^,^69^ to identify the most central parts of signaling networks. One of the most straightforward investigations was carried out by Kahn and colleagues^8^. They defined the critical nodes in the insulin pathway as a group of similar proteins that are elements of cross-talk and essential in insulin signal propagation. Alternative options to find essential proteins are network centrality measurements. Here the interconnected signaling pathways are represented as graphs, and various centrality measures are used to predict essential/critical proteins^65^,^66^,^67^. Graph-representation approaches depend on our *a priori* knowledge of the network. To prevent such caveats most of the studies that found essential proteins added biological information to the signaling or protein-protein interaction networks. Biological information could be differential expression^70^ or the hypothesis (which was used in the studies of Luo *et al*.^68^,^69^ and Li *et al*.^66^) that if a protein was a member of a protein complex, it would have a higher chance to be essential.

In the current work we combined Kahn and colleagues’ concept^8^ with tissue-specific network analysis to identify and analyze critical paralog groups in the human signaling network. We formed paralog groups based on two complementary resources. We generated tissue-specific networks using an expression-based dataset to check which protein is essential to connect different ligands to transcription factors. Then we tested whether a member of the paralog group is involved in cross-talk between pathways. To conduct the analysis we used SignaLink 2, a cross-talk specific signaling network resource^13^. With this workflow we found 75 critical paralog groups (CPGs) containing 267 critical proteins (CPs) in seven human signaling pathways (Fig. 1b). In the previous section – through three examples – we demonstrated the major mechanisms of signaling flow regulation by CPGs illustrating the structural and regulation-based mechanisms of certain CPGs (Fig. 5).

CPGs differ from non-CPGs because they are not only more central in the network (Fig. 2a) but also have a higher range of centralities (Fig. 2b), and have more biological functions in general, as well as more paralog-specific biological functions (Fig. 3b). These results are in good agreement with the literature in the sense that higher degree nodes (hubs) are essential^28^ and have more diverse biological functions^71^. The paralog groups by definition contain similar proteins to each other. Similar proteins with similar functions are the seeds of adaptation and evolutionary innovation^72^,^73^. An example of the evolution of different functions can be seen in the SMAD2 and SMAD3 pair (detailed in the Results section; Fig. 5a).

After a gene duplication event, regulation of the corresponding proteins (paralogs) can also start to evolve. We found that CPGs are regulated by more diverse miRNAs than non-CPGs (Fig. 3b). This finding is supported by other research; central proteins have more biological function and are regulated more strictly^28^,^74^. Interestingly, CPs have more specific post-transcriptional regulation, meanwhile the transcriptional regulation is similar to paralog proteins. A possible reason behind this phenomenon is that the miRNA regulatory network could be more able to evolve than the transcriptional regulatory network^75^. MiRNAs are fine-tuners of signaling network after the transcription^76^ therefore, miRNAs can set the proper ratio of paralogs within a paralog group to achieve paralog-specific signaling routes and biological functions. We showed how important this regulation can be in the example of MAPK signaling in the Results section (Fig. 5c). Another possibility of regulation may be achieved using different phosphorylation sites such as in the example of the SMAD2 and SMAD3 critical paralog group (Fig. 5a) or using different cofactors such as in the example of the NOTCH critical paralog group (Fig. 5b).

Mutations associated to inherited diseases and to cancer drivers are more common among critical proteins than within paralog proteins generally (Fig. 4a,d). Associated diseases are more specific in the case of different CPs within a CPG than in the case of different PPs in a non-CPG (Fig. 1c). In signaling the cancer driver mutations and mutations involved in inherited diseases are common^77^,^78^. Thus, the background distribution of these mutations is high in our dataset. In light of the high background mutation frequency it is surprising that PPs have fewer cancer driver mutations and inherited disease mutations than other proteins and critical paralogs (Fig. 4). The similar function and network centrality of PPs within a paralog group (Figs 2b and 3b) could explain this phenomenon whereby the paralogs in a non-CPG can take over each other’s function. Due to this redundancy a signaling related disease has to alter each paralog within a non-CPG to change the signaling flow, meanwhile it might be enough to alter only a single CP or other protein to achieve a disease phenotype (Fig. 1c). With our methods, we could distinguish between the disease causing critical paralogs and the redundant paralog proteins.

Our study distinguished two different kinds of paralogs to predict the effect of drugs. There are non-critical paralog groups in which members are similar to each other, have similar effects on the network (Fig. 2b) and have similar biological processes (Fig. 3b). Critical paralog groups have different and specific effects on networks, have specific biological roles and cause different diseases (Figs 2b, 3b and 4b). Drug discovery efforts should concentrate on critical proteins and not on paralog proteins.

Despite the care that we have taken to compile the datasets for our analyses, there are a few possible sources of biases and limitations in our study. Our work may be affected by the database of choice. To the best of our knowledge SignaLink 2 has the most straightforward pathway and cross-talk definition^13^ but our analysis should be tested in the future using other pathway curation sources like Reactome^79^ or Signor^80^. Information about cross-talk proteins is the determining factor in the analysis of other datasets because if a protein from a paralog group was involved in a cross-talk then the group was counted as a critical paralog group. Signaling pathway annotations in Reactome and Signor are not based on evolution and biochemical criteria, and both contain many small pathways like “IL1 signaling”, “IL6”, meanwhile Signor also contain large biological processes like “mitochondrial control of apoptosis” or “osteogeneisis”. Signor contains a significant portion of the SignaLink 2 database. The paralog group prediction depends on the resources used. We tried here to overcome bias by using two sources (OrthoDB and InParanoid^15^,^16^). For the biological function analysis we used Gene Ontology^81^. The depth of Gene Ontology terms depends strongly on how well studied a protein is. The network hubs are often more studied, therefore they appear to be involved in more biological processes^82^. This bias is present in any manually annotated pathway or biological database^82^. As not all proteins listed in SignaLink 2 had tissue-specific annotation, we also conducted an analysis incorporating the unannotated proteins to every tissue (generating potential *false positives* with lesser known proteins but decreasing *false negatives*). Although, it did not matter if unannotated proteins were excluded or included as the same critical paralog groups and critical proteins emerged (see Materials and Methods). It is important to note that our analysis is transferable to any tissues, pathogenic conditions, or cell lines, for which expression data is available to find CPGs under given conditions. The workflow of the systematic identification process and the presented analysis can be easily reproduced if any applied databases are updated.

To conclude, we identified and analyzed critical paralog groups in seven major pathways in humans and demonstrated how critical paralog groups can regulate signaling flow on a systems-level. First we used two different databases to construct paralog groups involved in signaling which were used to annotate proteins to signaling pathways. Then we constructed a workflow to find the critical paralog groups (paralog groups with have cross-talking paralogs, and members are essential in a tissue-specific signaling flow) to distinguish them from non-critical paralog groups. We needed only two criteria to distinguish the critical and non-critical paralog groups: tissue-specific information flow through a member of a paralog group and cross-talk between signaling pathways (Fig. 1a). Critical paralog groups and their members were found to be important in the pathological rewiring during diseases such as cancer and relevant in drug discovery, unlike the non-critical paralog groups members. As convincing examples, we showed the three major mechanisms that make critical paralog groups a source of signaling diversification (diversification in structure, paralog-specific regulation by co-factors and paralog-specific expression) and allow fine-tuning of the signaling network. We found indispensable, critical paralogs in the signaling network and distinguished them from the redundant paralogs aggregated to non-critical paralog groups. Our work could facilitate drug target selection and further studies in understanding disease pathogenesis.

## Additional Information

**How to cite this article**: Modos, D. *et al*. Identification of critical paralog groups with indispensable roles in the regulation of signaling flow. *Sci. Rep.* **6**, 38588; doi: 10.1038/srep38588 (2016).

**Publisher's note:** Springer Nature remains neutral with regard to jurisdictional claims in published maps and institutional affiliations.

## Supplementary Material

Supplementary Table 1

Supplementary Table 2

Supplementary Table 3
